# Erosive Resistance of High-Translucency Resin Composites: New Insights for Esthetic Dentistry

**DOI:** 10.4317/jced.63433

**Published:** 2025-11-30

**Authors:** José Giancarlo Tozo-Burgos, Wilfredo Escalante-Otárola, Marco Sánchez-Tito

**Affiliations:** 1 Research Group on Dental Biomaterials and Natural Products, Faculty of Health Sciences, Universidad Privada de Tacna, Tacna, Peru; 2 Universidad Catolica de Santa Maria, School of Dentistry, Arequipa, Peru

## Abstract

**Background:**

Acidic beverages can compromise the performance of resin composites by altering their mechanical properties. This study evaluated the effect of an energy drink on the microhardness and surface roughness of five high-translucency resin composites.

**Material and Methods:**

An in vitro experimental design was performed using Filtek Z350 XT Translucent, Forma Trans, PALFIQUE LX5 CE, Beautifil II Inc, and Opallis T-Neutral. Disc specimens (n = 5 per group) were light-cured with an LED unit and polished. The samples were immersed daily for 10 minutes in Red Bull® over 30 days and stored in artificial saliva between immersions. Microhardness (VMH) was measured using a 50 g load applied for 15 s, while surface roughness (Ra) was determined with a contact profilometer. Normality and homogeneity were assessed with Shapiro-Wilk and Brown-Forsythe tests. Two-way repeated-measures ANOVA with Bonferroni correction and Kruskal-Wallis with Dunn's post hoc were applied ( = 0.05).

**Results:**

All composites exhibited a progressive decrease in microhardness and an increase in surface roughness. Filtek Z350 XT Translucent maintained the highest hardness, whereas Forma Trans demonstrated the greatest stability with less deterioration. Beautifil II Inc showed the most pronounced changes in both variables, while PALFIQUE LX5 CE displayed balanced behavior and Opallis T-Neutral showed intermediate results. Statistically significant effects of resin type, immersion time, and their interaction were observed for both VMH and Ra (p&lt;0.001).

**Conclusions:**

In conclusion, daily exposure to Red Bull® negatively affected all resin composites tested. Beautifil II Inc proved the most susceptible, while nanocluster and zirconia containing materials exhibited greater resistance to acidic degradation.

## Introduction

In recent years, esthetic dentistry has gained prominence due to the increasing demand for minimally invasive treatments that meet high aesthetic standards. As a result, therapeutic approaches have shifted towards additive interventions, with resin composites becoming the preferred treatment option. These materials offer several advantages, including excellent esthetics, preservation of tooth structure, and accessibility (1 - 3). The dental market provides a diverse range of resin composites, with high-translucency composites emerging as the primary choice for anterior restorations. This preference is due to their ability to replicate the natural translucency of teeth and achieve harmonious aesthetic outcomes (4 - 6). Currently, only a limited number of studies have focused on high-translucency composites, primarily examining their optical properties. Piccoli et al. (7) evaluated the stability of these composites after exposure to water and coffee, finding that while some maintained their translucency better than others, products like IPS Empress Direct and Opallis T-Neutral exhibited greater opacification. The unique clinical behavior of high-translucency composites can be attributed to their composition, which differs from conventional resin composites. According to Bauer and Ilie (8), these translucent composites contain a lower proportion of inorganic fillers and a higher proportion of the organic matrix. This characteristic, besides enhancing translucency, may also affect their physical and mechanical properties, highlighting the need for more comprehensive analysis to understand their clinical performance. Assessing the impact of commonly consumed beverages on the physical and color stability of resin composites is crucial for predicting their performance under external challenges, both in controlled experimental settings and clinical practice (9). Several studies have indicated that components like phosphoric acid and carbon dioxide in carbonated beverages, ethanol in alcoholic drinks, and tannins and polyphenols in coffee and black tea can adversely affect the organic matrix of restorative materials, leading to surface erosion and progressive degradation (10 - 13). Recently, energy drinks have drawn significant scientific interest due to their rising popularity, particularly among children and adolescents, who often view them as products that enhance physical and mental performance (14 , 15). Energy drinks primarily contain caffeine in varying concentrations, along with other ingredients such as taurine, B-complex vitamins, vitamin C, niacin, and pantothenic acid (16). These components have been linked to erosive effects on dental enamel and have shown to alter surface properties such as microhardness, surface roughness, and mass loss in various restorative materials, including conventional resin composites, bulk-fill resins, and bioactive materials like resin-modified glass ionomers (17 - 21). Although several high-translucency resin composites are currently available on the market, to our knowledge, the scientific literature on them remains limited. While previous research has demonstrated that certain beverages negatively impact the properties of conventional composites, it is unclear whether these effects are comparable in high-translucency composites, which have distinct internal structures with varying resin matrix compositions, filler types, and sizes. Therefore, the aim of this study is to evaluate the microhardness and surface roughness of five high-translucency resin composites exposed to a widely consumed energy drink.

## Material and Methods

- Study design, ethical aspects, and sample size This in vitro experimental study received approval from the Ethics Committee of the Faculty of Health Sciences at Universidad Privada de Tacna, Peru (approval number: 141-08-2025). Five experimental groups were established to assess the effects of an energy drink on the surface roughness and microhardness of high-translucency resin composites under various storage conditions. Sample size calculation was performed using G*Power 3.1.3 (Heinrich Heine Universität, Düsseldorf, Germany), applying a one-way fixed-effects ANOVA, with a statistical power of 0.8, an alpha error of 0.05, and an effect size of 1.03 derived from the pooled standard deviation data of a previous study (22). The total calculated sample size was 20 specimens, achieving a statistical power of 90.6%. The specimens were randomly assigned to five groups (n=5) using Research Randomizer, version 4.0 (Social Psychology Network, Middletown, CT, USA). The resins selected for this study correspond to those available on the Peruvian market; specific details such as brand, shade, batch, and composition are provided in Table 1.

Table 1

- Specimen preparation Five discs per group were fabricated using a metallic mold with an internal diameter of 8 mm and a thickness of 1.5 mm (23). The resin composite was placed in a single increment and covered with a Mylar strip. A glass slide was then used to flatten the composite, ensuring a smooth surface. The specimens were light-cured for 20 s (24), using a LED curing unit (VALO Cordless, Ultradent Products Inc., South Jordan, UT, USA) operating at 1200 mW/cm². After curing, both surfaces of the specimens were polished using the Super-Snap polishing system (Shofu Inc., Kyoto, Japan), following the sequential use of color-coded abrasive discs: black (coarse), violet (medium), green (fine), and red (superfine), applied with light pressure and continuous movements for approximately 30 s each. The final thickness of the specimens was measured with a digital caliper (IP65 Digital Micrometer, Asimeto®, Mooresville, NC, USA). Finally, all samples were stored in deionized water until testing (25). - Microhardness evaluation Vickers microhardness values were measured using a microhardness tester (AMH55; LECO, St. Joseph, MI, USA). Three indentations were made on each specimen, spaced 0.5 mm apart, with a load of 50 g applied for 15 s. Measurements were taken at three intervals: before immersion, after 7 days of immersion, and after 30 days of immersion (19). - Surface roughness evaluation Surface roughness was assessed using a contact profilometer (SJ-210; Mitutoyo America Corporation, Aurora, IL, USA). Three measurements were recorded at different locations on each specimen, tracing a distance of 2 mm, with a load of 4 mN and a traversing speed of 0.5 mm/s. A cutoff value (c) of 0.25 mm was applied. The main parameter recorded was the average Ra value (2). Measurements were conducted at three intervals: before immersion, after 7 days of immersion, and after 30 days of immersion (19). - Immersion protocol The pH of Red Bull® (Red Bull GmbH, Fuschl am See, Austria) was measured in triplicate at room temperature using a calibrated digital pH meter (BIOBASE®, PH-25C; Jinan, Shandong, China). For titratable acidity, 50 mL of the energy drink were transferred into a beaker and maintained under magnetic stirring without heating. Aliquots of 0.2 mL of 1.0 M NaOH were added incrementally until a stable pH of 5.5 was achieved (17). Each specimen was individually immersed in 20 mL of Red Bull® at room temperature. The resin discs were immersed for 10 min once daily for a total of 30 consecutive days. Between daily immersions, the samples were stored in artificial saliva at 37°C to simulate intraoral conditions. - Scanning electron microscopic (SEM) analysis For surface morphology analysis, one representative specimen from each group was selected. The samples were mounted on double-sided carbon adhesive tape (Electron Microscopy Sciences, Washington, PA, USA) and sputter-coated with gold-palladium for 30 s in a vacuum metallizing unit (Sputter Coater SC7620; Quorum Technologies Ltd., Laughton, East Sussex, UK). A scanning electron microscope (EVO 10 MA; Carl Zeiss Microscopy GmbH, Jena, Germany) was used to examine the microstructure of the specimens at 200× magnification, with a working distance of 7 mm and an accelerating voltage of 8 kV. Observations were performed using a secondary electron detector in high-vacuum mode. - Statistical Analysis Data analysis was conducted using GraphPad Prism software, version 10.0 (GraphPad Software, San Diego, CA, USA). The Shapiro-Wilk test and the Brown-Forsythe test were utilized to assess normality and homogeneity of variance, respectively. A two-way repeated measures ANOVA was performed, applying Bonferroni correction for multiple comparisons. Differences in microhardness and surface roughness values of the translucent composites at T0-T1 and T0-T2 were analyzed using the Kruskal-Wallis test, followed by Dunn's post hoc test. A significance level of 5% was set for all statistical tests.

## Results

- pH and titratable acidity The pH evaluation showed a value of 3.1 for Red Bull®. Regarding titratable acidity, a value of 3.7 mL was recorded at pH 5.5. - Microhardness and surface roughness analyses Table 2 summarizes the mean Vickers microhardness (VMH) and surface roughness (Ra) values of each resin composite at the different immersion times in the energy drink.

Table 2

**Figure 1:**
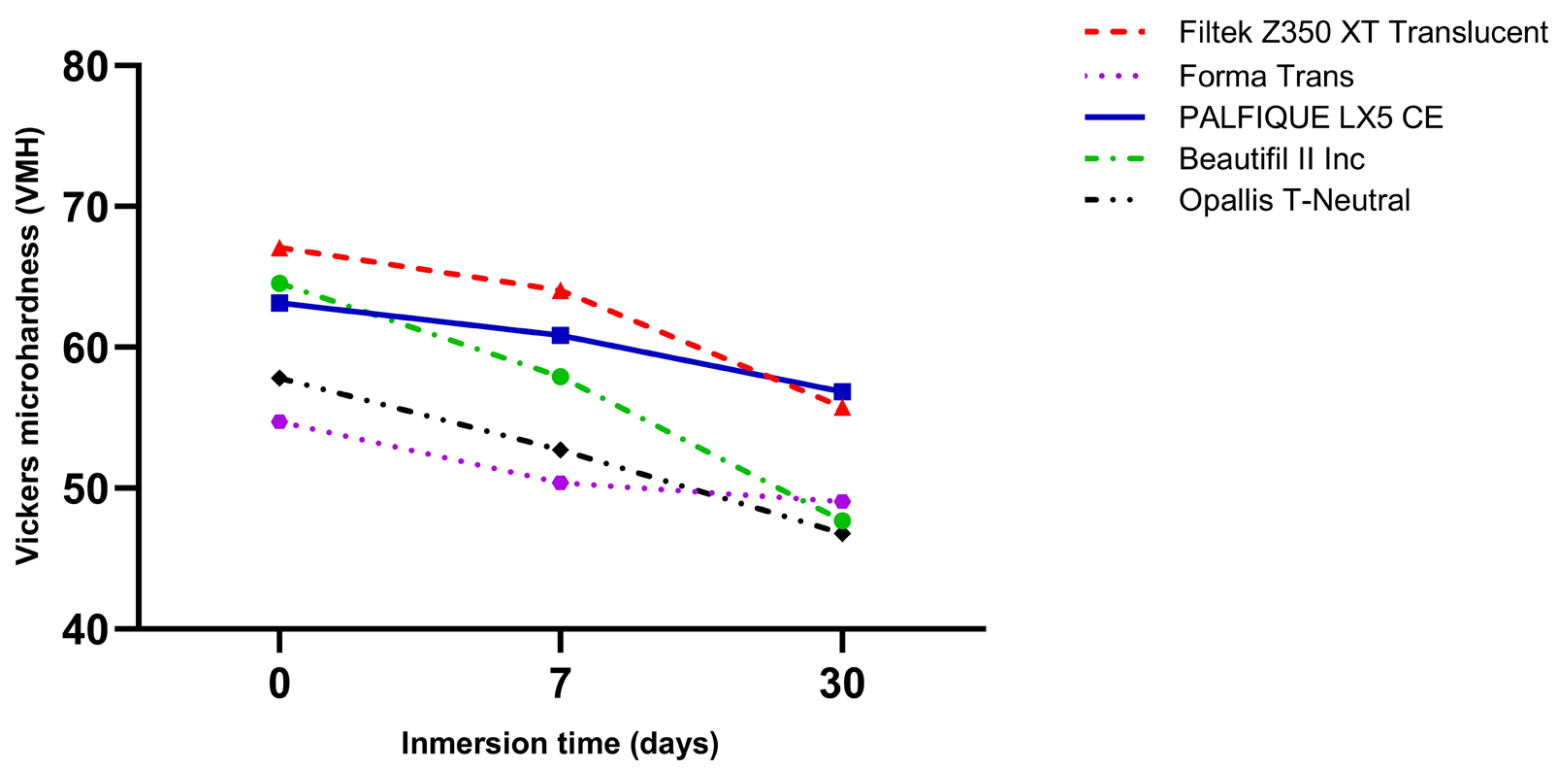
Vickers microhardness of high-translucency resin composites after immersion in Red Bull® at 0, 7, and 30 days.

Throughout the 30-day immersion period, all resin composites demonstrated a gradual decrease in VMH, coupled with a corresponding increase in Ra. Filtek Z350 XT Translucent consistently maintained the highest microhardness values at all evaluation points, followed by Beautifil II Inc and Opallis T-Neutral. In contrast, Forma Trans displayed the lowest VMH values throughout the study. Notably, Beautifil II Inc exhibited the greatest reduction in microhardness at day 30 (Fig. 1).

1

**Figure 2:**
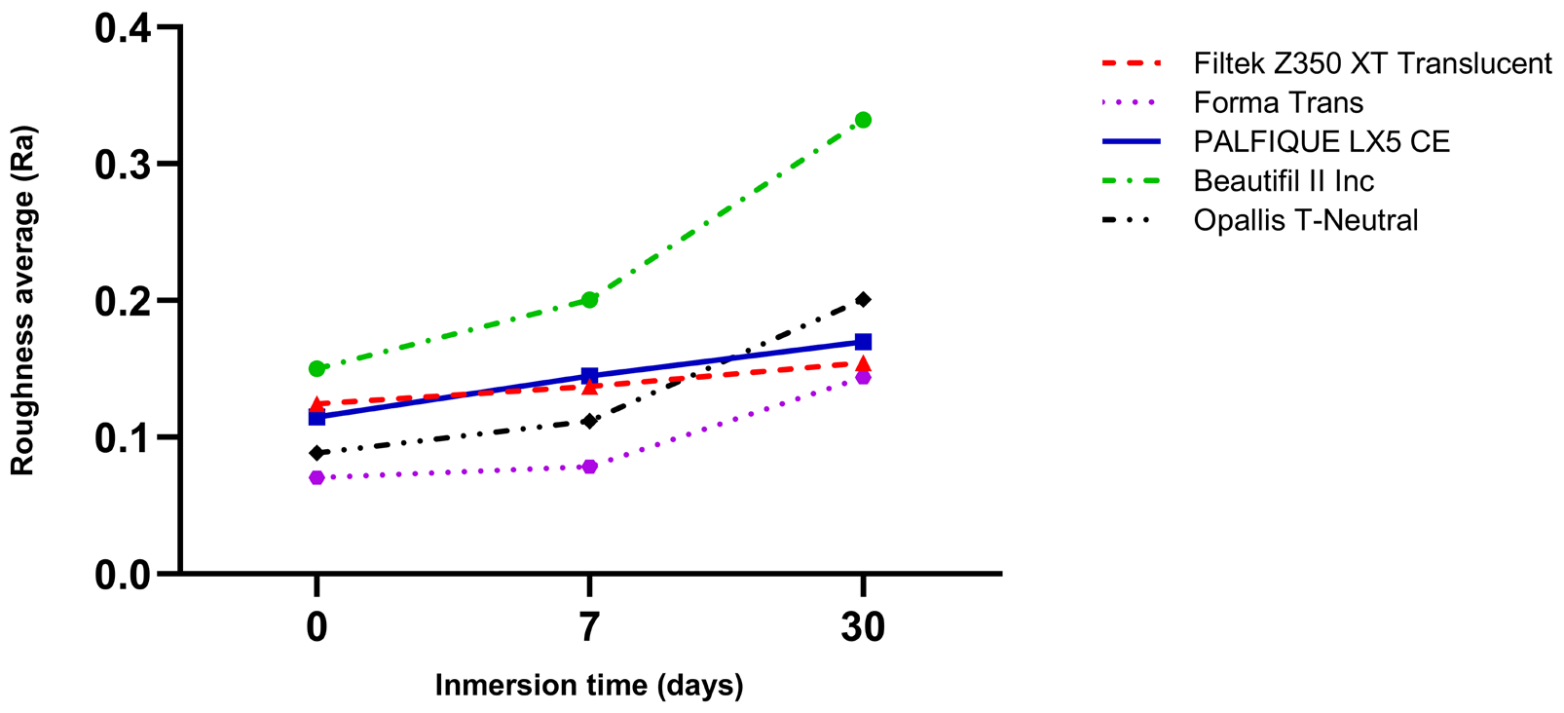
Surface roughness of high-translucency resin composites after immersion in Red Bull® at 0, 7, and 30 days.

Regarding surface roughness, all resins exhibited rising Ra values over time, with Beautifil II Inc showing the most substantial increase, especially between days 7 and 30. Forma Trans and Filtek Z350 XT Translucent displayed more stable behavior with the smallest changes in roughness, while PALFIQUE LX5 CE and Opallis T-Neutral exhibited intermediate trends (Fig. 2).

2

Table 3 presents the results of the two-way repeated measures ANOVA, which indicated statistically significant effects of both resin type and immersion time on microhardness (VMH) and surface roughness (Ra). For microhardness, significant differences were found among the resin groups (p&lt;0.001) and across different immersion times (p&lt;0.001).

Table 3

Additionally, there was a significant interaction between resin type and immersion time (p=0.004), suggesting that microhardness varied based on both factors. Similarly, surface roughness was significantly affected by resin type (p&lt;0.001) and immersion time (p&lt;0.001), with a significant interaction effect (p&lt;0.001) as well. Overall, the statistical models accounted for a substantial portion of the variability observed, explaining approximately 84.9% of the variance in microhardness (VMH) and 82.8% of the variance in surface roughness (Ra), as detailed in Table 3. The nonparametric Kruskal-Wallis test, followed by Dunn's post hoc comparisons, showed statistically significant differences in both microhardness (VMH) and surface roughness (Ra) among the resin composites over time. For VMH, no significant differences were found among the groups from T0 to T1 (p&gt;0.05); however, significant differences were noted from T0 to T2 (p=0.045). Beautifil II Inc demonstrated the greatest reduction in microhardness (16.86 ± 8.69), followed by Filtek Z350 XT Translucent (11.35 ± 3.05) and Opallis T-Neutral (11.02 ± 4.77). In contrast, Forma Trans and PALFIQUE LX5 CE exhibited smaller changes in microhardness (5.67 ± 4.44 and 6.29 ± 2.65, respectively) (Table 4).

Table 4

Regarding surface roughness, all resins exhibited negative Ra values, indicating an overall increase in surface roughness over time. At 7 days, Filtek Z350 XT Translucent and Forma Trans showed the smallest changes (Ra close to zero), while Beautifil II Inc presented the highest roughness increase. At 30 days, Filtek Z350 XT Translucent maintained the most stable behavior with the lowest Ra (-0.029), whereas Beautifil II Inc and Opallis T-Neutral demonstrated the greatest deterioration. PALFIQUE LX5 CE and Forma Trans showed intermediate values (Table 5).

Table 5

**Figure 3:**
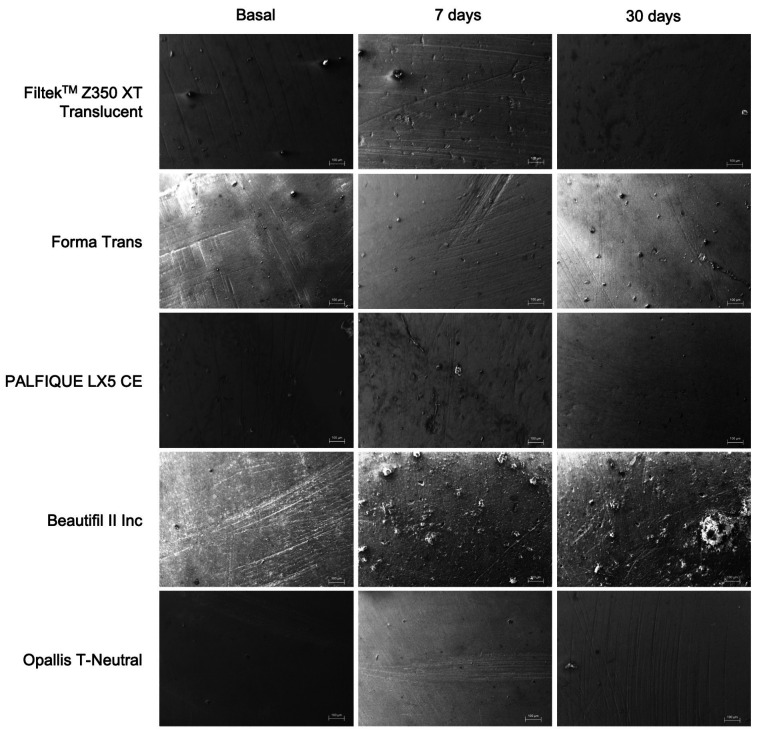
Scanning electron microscope evaluation of surface alteration in high-translucency resin composites over time.

- Qualitative analysis - SEM The images obtained through scanning electron microscopy (SEM) revealed superficial morphological changes caused by immersing the materials in the energy drink. In the Beautifil II Inc specimens, significant structural changes were noted after 7 and 30 days of exposure to Red Bull®, characterized by material loss and an irregular surface topography (Fig. 3).

[caption id="attachment_1810" align="alignnone" width="300"]3 Screenshot[/caption]

In contrast, the Filtek XT Translucent and Forma Trans specimens maintained more uniform surfaces, exhibiting minimal irregularities. Meanwhile, Opallis T-Neutral and PALFIQUE LX5 CE displayed intermediate changes, with visible signs of surface degradation, though this was less pronounced than the alterations observed in Beautifil II Inc.

## Discussion

High-translucency resin composites typically contain about 70-78 wt% inorganic fillers, which is slightly lower than the filler content in heavily filled composites. This is accompanied by a proportional 20-30% organic matrix. This specific composition enhances light transmission, contributing to their high translucency, although it may also impact their mechanical properties (8). The results of this study demonstrated a gradual decrease in microhardness values for all high-translucency resins after 7 and 30 days of immersion in an energy drink. This was assessed using a protocol that involved 10 min of immersion per day to simulate the average consumption time and intraoral retention, as established in previous studies (19). This investigation confirmed that certain solutions have a significantly harmful effect on the surface of resins, as evidenced by the gradual increase in surface roughness (Ra), indicating notable topographical degradation. These findings align with those reported by Erdemir et al. (22) and Loo-Valle et al. (19), who demonstrated that energy drinks lead to the softening of the matrix and the development of surface irregularities in both conventional and bulk-fill composites. Their studies noted significant reductions in microhardness under similar experimental conditions. The detrimental effects have been attributed to the low pH and high acidity of these beverages, which facilitate the degradation of the organic matrix, weaken the matrix-filler interface, and disrupt the polymer network (26). Accordingly, the pH and titratable acidity values obtained for Red Bull® in this study were consistent with those reported in a previous investigation (17). Shenoy et al. (27) reported an initial pH and a titratable acidity value of 17.5 mL of NaOH required to raise the pH to 5.5. This difference can be explained by the fact that they used a 0.1 M NaOH solution, whereas in our study a 1.0 M concentration was employed, as reported in a previous investigation. Although High-translucency resins have a unique composition, featuring a higher organic fraction and lower filler content. The current results confirm that these materials, like other resin composites exposed to erosive environments, experience a loss of mechanical properties and an increase in surface roughness. This degradation may be more pronounced in translucent resins due to their specific composition. In terms of microhardness, Filtek Z350 XT Translucent consistently demonstrated the highest absolute values at all evaluation periods. This can be attributed to its unique filler composition, which includes larger silica and zirconia nanoclusters ranging from 0.6 to 20 µm. In contrast, the Dentin, Enamel, and Body variants possess nanoclusters that only range between 0.6 and 10 µm (28). On the other hand, Forma Trans displayed lower baseline microhardness values but showed the most stable surface performance, with a less pronounced reduction in microhardness (5.67 ± 4.44 VMH). This stability may be due to its filler system, which comprises zirconia and ytterbium trifluoride (YbF3) particles. The uniform morphology of these particles allows for a homogeneous distribution within the matrix, thereby minimizing filler leaching in acidic environments (29 , 30). In contrast, Beautifil II Inc demonstrated the most significant reduction in microhardness, with a substantial difference between baseline and final measurements (16.86 ± 8.69). This behavior can be attributed to the presence of S-PRG fillers, which are designed to provide bioactivity through ion release, supporting remineralization and antimicrobial activity. However, Pimentel et al. (31) pointed out that these same characteristics can increase the permeability of the matrix in acidic environments, such as low-pH energy drinks. These drinks can infiltrate the matrix-filler interface, leading to polymer matrix hydrolysis and ion leaching. As a result, there is an accelerated degradation of the organic phase and a progressive weakening of the bond between the matrix and fillers, causing a significant loss in microhardness. Therefore, resins containing S-PRG are more vulnerable to physical degradation under erosive conditions since the active and permeable structure of these fillers allows for acid penetration and the breakdown of both the matrix and interface, leading to faster deterioration compared to conventional resins. Opallis T-Neutral displayed intermediate performance, exhibiting less microhardness loss than Beautifil II Inc., but more than Forma Trans. This microhybrid composite has a matrix composed of Bis-GMA, Bis-EMA, UDMA, and TEGDMA, combined with an inorganic filler made of Schott glass that has an average particle size of 0.5 µm. The filler content reaches approximately 78-79 wt%. This ratio and distribution of filler seem to provide moderate resistance to erosive challenges (32). PALFIQUE LX5 CE demonstrated a distinct performance; while it did not achieve high initial microhardness values, it maintained remarkable stability throughout the experimental period, with an average of 6.29 ± 2.65. This stability can be attributed to its high filler content of 82 wt% and 71 vol%. The filler consists of uniformly distributed supra-nano spherical silica and zirconia particles within a Bis-GMA/TEGDMA-based resin matrix (33). This configuration minimizes filler loss in acidic conditions, and the efficient coupling between the matrix and filler, along with the uniformity of the filler morphology, likely limits degradation. Thus, this explains its stable behavior despite lower absolute microhardness values compared to other resins. The most stable resin was Filtek Z350 XT Translucent, displaying only a slight change in final Ra values (-0.029 ± 0.018), Forma Trans also performed well, showing a change of -0.073 ± 0.511. However, comparisons with previous studies are limited because it has not been tested under erosive conditions. PALFIQUE LX5 CE and Opallis T-Neutral exhibited moderate changes, which were still smaller than those seen in Beautifil II Inc. The latter demonstrated the most significant increase in roughness, indicating a greater susceptibility to acidic environments. Scanning electron microscopy (SEM) provided further insight into the surface alterations induced by immersion in the energy drink. The micrographs confirmed the roughness trends, showing that Beautifil II Inc exhibited the most evident changes, characterized by material loss and a markedly irregular surface. In contrast, Filtek Z350 XT Translucent and Forma Trans maintained more uniform surfaces with minimal irregularities, consistent with the stability observed in their Ra values. These qualitative findings provide a visual dimension that reinforces the interpretation of surface deterioration in the tested resins. In this study, Red Bull® was chosen as the erosive medium due to its low pH and high acidity, characteristics that have been linked to the deterioration of the mechanical and surface properties of restorative materials. Several investigations have shown that energy drinks, including Red Bull®, can increase surface roughness and decrease the microhardness of resin composites. Al-Samadani (34) reported that the consumption of Red Bull® and Monster led to progressive surface deterioration of nanofilled composites, as evidenced by a gradual increase in roughness after six months of exposure. Similarly, Tanthanuch, et al. (20) found that sports and energy drinks reduced hardness and increased roughness in various restorative materials, including bioactive composites. Overall, these findings confirm the significant impact of such beverages on the physical properties of restorative materials, which supports the selection of Red Bull® as the erosive agent in this study. As previously mentioned, to our knowledge, no studies have specifically addressed the behavior of highly translucent composite resins, such as those examined in this analysis, regarding microhardness, surface roughness, or other physical and mechanical properties under erosive conditions. Most available literature has focused on different aspects. Piccoli et al. (7) demonstrated that Filtek Z350 XT Translucent and Opallis T-Neutral maintained adequate optical stability after exposure to staining agents. In contrast, Pimentel et al. (31) found that materials containing S-PRG, like Beautifil II Inc, are more susceptible to physical degradation under erosive conditions. However, there are currently no reports addressing the physical and mechanical stability of these translucent composites specifically in relation to exposure to energy drinks. In this regard, the present findings provide valuable evidence on the microhardness and surface roughness of this group of resins, contributing to a better understanding of their clinical performance in highly erosive environments. Among the limitations of this study, it is important to acknowledge its in vitro design, which does not fully replicate the complex dynamics of the oral cavity. Oral factors such as salivary flow, pH fluctuations, the acquired pellicle, biofilm, and the buffering effect of saliva are not reproduced. In addition, only two mechanical properties were evaluated, so the findings cannot be directly extrapolated to all clinical conditions. Moreover, although only one energy drink was tested, Red Bull® is one of the most widely marketed beverages worldwide, and its formulation is comparable to that of other brands. Finally, the immersion period employed in this study approximates habitual consumption but does not accurately reflect the frequency, volume, or possible interactions with other dietary habits of patients. Further studies should consider larger sample sizes, to confirm the present findings despite the high statistical power achieved with the current design.

## Conclusions

All high-translucency resin composites evaluated showed a decrease in microhardness and an increase in surface roughness after being exposed to the energy drink. Among the materials tested, Forma Trans exhibited the greatest stability in microhardness, while Filtek Z350 XT Translucent maintained surface roughness more effectively. PALFIQUE LX5 CE demonstrated a balanced performance, achieving favorable results in both parameters, whereas Opallis T-Neutral displayed intermediate characteristics. In contrast, Beautifil II Inc proved to be the least stable for both properties, highlighting the greater vulnerability of giomers when exposed to acidic environments.

## Figures and Tables

**Table 1:** High-translucency resin composites used in this study.

Resin Composite	Shade	batch	Origin	Characteristics
Filtek™ Z350 XT Translucent CT (3M)	Clear Translucent	11118878	United States (St. Paul, Minnesota)	Resin matrix composed of Bis-GMA (bisphenol A-glycidyl methacrylate), UDMA (urethane dimethacrylate), TEGDMA (triethylene glycol dimethacrylate), and Bis-EMA (ethoxylated bisphenol A dimethacrylate). Contains 20 nm silica nanoparticles and silica/zirconia nanoclusters (0.4-0.6 µm). Inorganic filler loading: 78.5 wt%.
Forma Trans (Ultradent)	Transparent	D0NYF	United States (South Jordan, Utah)	Nano-hybrid composite formulated with an organic matrix based on Bis-GMA, UDMA, TEGDMA, and Bis-EMA, and inorganic fillers of zirconia and ytterbium trifluoride with particle sizes ranging from 0.014 to 3 µm; filler content approx. 72-75 wt% and 25-28 wt% organic matrix.
PALAFIQUE LX5 CE (Inc.) (Tokuyama)	CE (Inc.)	W5371	Japan (Tokyo)	Resin composite with 82 wt% (71 vol%) filler composed of silica-zirconia and composite filler. Incorporates submicron spherical particles with an average size of 0.2 µm.
Beautifil II Inc. (Shofu)	INC (Incisal)	042473	Japan (Kyoto)	Bioactive nano-hybrid composite incorporating S-PRG (Surface Pre-Reacted Glass-ionomer) technology, allowing fluoride release and recharge. Contains discrete nano-fillers (10-20 nm), with a filler loading of 83.3 wt%.
Opallis T-Neutral (FGM)	T-Neutral	170622	Brazil (Joinville, Santa Catarina)	Nano-hybrid composite formulated with Schott glass particles and silica nanoparticles with an average size of 0.5 µm.

1

**Table 2:** Microhardness (VMH) and surface roughness (Ra) of resin composites according to immersion time (Mean ± SD).

Resin	Vickers microhardness			Surface roughness		
	Initial (T0)	7 days (T1)	30 days (T2)	Initial (T0)	7 days (T1)	30 days (T2)
Filtek Z350 XT Translucent	67.09 ± 3.76	64.04 ± 1.97	55.74 ± 2.59	0.124 ± 0.039	0.137 ± 0.025	0.154 ± 0.024
Forma Trans	54.72 ± 2.56	50.39 ± 4.94	49.04 ± 5.07	0.076 ± 0.023	0.078 ± 0.024	0.144 ± 0.065
PALFIQUE LX5 CE	63.16 ± 4.30	60.83 ± 4.74	56.86 ± 6.73	0.114 ± 0.052	0.145 ± 0.049	0.170 ± 0.081
Beautifil II Inc	64.54 ± 1.40	57.92 ± 2.85	47.68 ± 7.34	0.150 ± 0.027	0.200 ± 0.038	0.332 ± 0.061
Opallis T-Neutral	57.80 ± 2.77	52.72 ± 5.75	46.78 ± 6.83	0.088 ± 0.012	0.111 ± 0.007	0.201 ± 0.043

2

**Table 3:** Two-way repeated ANOVA results for Vickers microhardness (VMH) and surface roughness (Ra).

Parameter	Source	Sum of squares	df	Mean squares	F	p	Adj. R-squared
Microhardness	Model	3854.44	34	113.37	13.23	<0.001	0.849
	Resin	1358.11	4	339.52	7.25	<0.001	
	Time	1322.75	2	661.37	77.17	<0.001	
	Resin*Time	237.62	8	29.70	3.47	0.004	
Roughness	Model	0.362	34	0.010	11.49	<0.001	0.828
	Resin	0.137	4	0.034	9.10	<0.001	
	Time	0.109	2	0.054	59.00	<0.001	
	Resin*Time	0.040	8	0.005	5.42	<0.001	

3

**Table 4:** Comparison of differences in Vickers microhardness (ΔVMH) values between resins.

Resin	T0-T1		p-value*	T0-T2		p-value*
	Mean ± SD	Median [IQR]		Mean ± SD	Median [IQR]	
Filtek Z350 XT Translucent	3.04 ± 2.29	2.80 [3.64]a	0.459	11.35 ± 3.05	12.23 [1.27]a	0.045
Forma Trans	4.32 ± 3.54	4.43 [5.67]a		5.67 ± 4.44	4.10 [3.00]bde	
PALFIQUE LX5 CE	2.31 ± 1.37	2.13 [0.50]a		6.29 ± 2.65	6.17 [4.83]cdf	
Beautifil II Inc	6.62 ± 3.60	6.50 [3.43]a		16.86 ± 8.69	16.90 [11.66]a	
Opallis T-Neutral	5.08 ± 4.21	7.13 [7.26]a		11.02 ± 4.77	10.90 [1.74]aef	

For each column, equal superscript letters indicate the absence of statistically significant differences among the resin composites (p>0.05); * Kruskal-Wallis test followed by Dunn’s test; SD: Standard deviation; IQR: Interquartile range

**Table 5:** Comparison of differences surface roughness (ΔRa) values between resins.

Resin	T0-T1		p-value*	T0-T2		p-value*
	Mean ± SD	Median [IQR]		Mean ± SD	Median [IQR]	
Filtek Z350 XT Translucent	-0.012 ± 0.015	-0.007 [0.012]a	0.005	-0.029 ± 0.018	-0.033 [0.005]a	0.005
Forma Trans	-0.008 ± 0.008	-0.009 [0.014]b		-0.073 ± 0.511	-0.052 [0.017]ac	
PALFIQUE LX5 CE	-0.030 ± 0.013	-0.027 [0.019]cef		-0.055 ± 0.036	-0.049 [0.058]a	
Beautifil II Inc	-0.050 ± 0.014	-0.047 [0.024]de		-0.182 ± 0.082	-0.218 [0.146]bd	
Opallis T-Neutral	-0.023 ± 0.010	-0.021 [0.011]abf		-0.112 ± 0.039	-0.103 [0.057]cd	

For each column, equal superscript letters indicate the absence of statistically significant differences among the resin composites (p>0.05); * Kruskal-Wallis test followed by Dunn’s test; SD: Standard deviation; IQR: Interquartile range

## Data Availability

The datasets generated and analyzed in this study are available from the corresponding author upon reasonable request.
